# Work-Related Stressors and Their Perceived Impact on Veterinary Work and Personal Life: A Multi-Country European Study

**DOI:** 10.3390/vetsci13060583

**Published:** 2026-06-15

**Authors:** Marietta Máté, Claire Helen Várnai, László Ózsvári

**Affiliations:** Department of Veterinary Forensics and Economics, University of Veterinary Medicine Budapest, István Street 2, 1078 Budapest, Hungary; clairevarnai@gmail.com (C.H.V.); ozsvari.laszlo@univet.hu (L.Ó.)

**Keywords:** burnout, client communication, emotional exhaustion, euthanasia, fatigue, mental health, veterinarians, well-being, work–life balance, work-related stressors

## Abstract

Work-related stress is an important concern in veterinary medicine because it may affect veterinarians’ work, personal life, and well-being. This study described self-reported work-related stressors and their perceived effects on professional and personal life among veterinarians from selected European countries. Between July 2021 and February 2022, an online questionnaire was completed by 724 veterinarians from Hungary, Finland, Sweden, Germany, Denmark, Estonia, and Norway. Participants were recruited through convenience sampling via online channels and professional veterinary networks. The questionnaire assessed 16 stressors, including fatigue, emotional exhaustion, burnout-related symptoms, fear of making mistakes, client expectations, and negative online comments. Mean Likert-scale scores were analyzed using one-way ANOVA and Pearson’s χ^2^ tests. Fatigue and emotional exhaustion were among the most burdensome internal stressors, with the highest mean score in the Hungarian sample (mean: 4.15 ± 1.05) and the lowest in the Finnish sample (mean: 3.68 ± 1.06; ANOVA: *p* < 0.0001). Euthanasia-related stress was rated lower in Finland (mean: 1.68 ± 0.83) and Sweden (mean: 1.88 ± 0.95) than in Germany (mean: 2.41 ± 1.17) and Hungary (mean: 2.64 ± 1.27; ANOVA: *p* < 0.0001). In Hungary, younger and female veterinarians reported greater sensitivity to several stressors. The findings are descriptive and exploratory rather than representative cross-country comparisons.

## Simple Summary

Veterinary work can be stressful and may affect both professional performance and personal life. This study examined how veterinarians from multiple European countries perceived different work-related stressors, such as fatigue, emotional exhaustion, burnout-related symptoms, fear of making mistakes, difficult communication with animal owners, complaints, and negative online comments. Responses were collected from 724 veterinarians in Hungary, Finland, Sweden, Germany, Denmark, Estonia, and Norway. Fatigue and emotional exhaustion were among the most commonly reported burdens. Stress related to euthanasia differed between countries and was rated as less burdensome in Finland and Sweden than in Germany and Hungary. In the Hungarian sample, younger veterinarians and women reported greater sensitivity to several stressors. As participation was voluntary and the samples were not nationally representative, the findings should be interpreted as descriptive. The results may help veterinary workplaces, professional organizations, and educators identify areas where better support, improved communication, and healthier working conditions are needed.

## 1. Introduction

The link between occupational stressors in veterinary work and the mental health of veterinarians has long been a concern in the profession. Beyond its impact on individual wellbeing, chronic occupational stress may also affect professional functioning, including clinical judgement and client communication, with potential consequences for patient safety and the quality of veterinary care. Veterinary patient safety research shows that medical errors and clinical incidents can cause substantial harm, highlighting the importance of addressing workplace conditions that contribute to stress and impaired performance [1,2]. Work–life balance is essential for maintaining mental wellbeing, and job satisfaction acts as a mediating factor [3]. Stressors in veterinary work can be broadly distinguished from internal manifestations of strain, rooted in personal well-being, such as fatigue (including physical and mental exhaustion) and emotional exhaustion, and external occupational stressors associated with the work environment and professional interactions, such as heavy workload, client expectations, complaints and ethical challenges. Pohl et al. identified long working hours and ethical dilemmas as major sources of stress among veterinary professionals [4]. Fatigue and emotional exhaustion have also been described as important manifestations of occupational strain and secondary traumatic stress with elements of burnout [5]. Chronic occupational stress is one of the most critical challenges in the veterinary profession, and burnout is generally conceptualized as an occupational phenomenon resulting from chronic workplace stress that has not been successfully managed, rather than as a primary stressor [6,7]. Burnout is characterized by emotional exhaustion, depersonalization, and reduced personal accomplishment [8]. Steffey et al. confirms that chronic workplace stress and burnout have significant implications for both mental health and professional performance in veterinary medicine [9]. Burnout-related symptoms, such as emotional exhaustion and depersonalization, may further reduce coping capacity, increase vulnerability to anxiety and depression, and strain workplace relationships [10,11].

Among external occupational stressors in veterinary medicine, high workload, demanding client expectations and frequent client complaints, and ethically challenging situations, including but not limited to end-of-life and euthanasia-related situations, are consistently identified as major sources of stress. Veterinarians often operate at the intersection of competing interests of animals and clients, which can result in ethical conflicts when emotional, financial or welfare considerations diverge. Repeated exposure to morally challenging decisions, especially euthanasia under emotionally charged circumstances involving strong owner–animal bonds, has been associated with moral distress, traumatic stress responses and reduced psychological well-being [12,13,14,15,16,17]. A cross-cultural study found that the perceived ethical burden of euthanasia varies substantially between countries and genders, and may decrease with increasing professional exposure, highlighting the role of cultural, educational and organizational contexts in shaping veterinarians’ experiences [18].

Several studies have shown that veterinarians have higher rates of suicidal ideation, anxiety and depressive symptoms than the general population [4,19,20,21,22,23,24]. Constant exposure to challenging situations such as interpersonal conflict, euthanasia and easy access to opioids may contribute to poor mental health and high suicide rates among veterinarians [25]. At the same time, veterinarians tend to have a less positive attitude towards mental illness than the general population [26].

Prolonged job stress, long working hours, overwork, pressure from managers and employers, frequent conflicts with clients or colleagues, or inadequate pay can lead to burnout and physical and emotional exhaustion. Burnout not only reduces productivity and lowers work morale but can also negatively affect the quality of patient care [27,28,29]. When examining work-related help-seeking behaviors, it is important to account for influencing factors such as age [30,31], gender [32,33,34], work ability and specialization [32,33,34] and marital status [35]. Identifying which stressors exert the strongest influence is essential for designing targeted interventions in veterinary workplaces. Cross-country comparisons may help identify modifiable organizational and policy-level differences in working conditions and support systems, thereby informing targeted interventions to safeguard both veterinarian wellbeing and the quality of veterinary care [2,36].

However, descriptive evidence from this region, particularly from Hungary and Northern European countries, remains limited regarding how specific work-related stressors are perceived to be related to veterinarians’ professional and personal lives. The aims of this study were (1) to conduct an exploratory assessment of veterinarians’ perceptions of how work-related stressors and strain-related factors were associated with their professional and personal lives, as well as their perceptions of workplace support for mental health, in Hungary, Finland, Sweden, Germany, and other Northern European countries, including Denmark, Estonia, and Norway, and (2) to examine how demographic and occupational factors, such as age, gender, weekly working hours, job position and holidays, are linked to these experiences in Hungary.

## 2. Materials and Methods

### 2.1. Study Design and Data Collection

The survey was carried out using an online questionnaire, which was preceded by a comprehensive literature review to explore the stressors present in the veterinary profession and their impact on work and personal life. Based on the literature review, the first version of the questionnaire was developed and revised several times to ensure full coverage of relevant topics and issues. The original version was created in Hungarian and later translated into English. The translation process was conducted collaboratively within the research team, including a bilingual co-author with native-level proficiency in both Hungarian and English. Any inconsistencies or ambiguities in translation were reviewed and resolved by consensus to ensure conceptual and linguistic equivalence between the two versions. Before finalization, a pilot version of the questionnaire was tested among a group of 8 Hungarian veterinarians to assess clarity, relevance and comprehensibility. Feedback from these participants led to minor wording adjustments. No formal psychometric validation of the questionnaire was performed. The pilot testing was intended to improve the clarity and practical applicability of the questionnaire items and to support conceptual consistency between the Hungarian and English versions.

The finalized questionnaire (Supplementary Questionnaire), along with a brief description, was distributed to veterinarians in Hungary, Finland, Sweden, Germany, Estonia, Denmark and Norway. The veterinarians were informed that participation was voluntary and their responses would remain anonymous. All respondents gave their prior written informed consent electronically to the collection of data. Participants were recruited via convenience sampling through professional veterinary digital networks, closed online platforms, mailing lists, and veterinarian-only social media channels to efficiently reach a large and geographically diverse group of practitioners working in structurally and geographically diverse veterinary work environments across Central and Northern Europe. Due to the exploratory nature of the study, no a priori sample size calculation was conducted; instead, the aim was to reach as large and diverse a sample as possible within the survey period. Eligibility required a veterinary degree and current or previous employment in the veterinary field. Incomplete questionnaires were excluded, and only fully completed questionnaires were included in the final analysis.

In Hungary, the questionnaire was shared within the “*Állatorvosok-Vets*” Facebook group. Finnish and Swedish veterinarians were primarily contacted via email, with invitations sent to individual veterinarians via the mailing lists of the Finnish and Swedish veterinary associations, targeting their active members. In Finland, the questionnaire was also circulated in a Finnish Vet Facebook group to increase the number of responses, while in Sweden, it was shared with veterinary groups and featured in the Swedish Veterinary Association (SVF) newsletter. In Germany, the questionnaire was sent to veterinary mailing lists, and responses from other Northern European countries (Estonia, Denmark and Norway) were collected through Swedish and Finnish postings. Thus, recruitment combined email invitations sent via professional associations’ mailing lists with closed social media groups for veterinarians, allowing broad coverage while ensuring that only qualified practitioners could participate. The survey opened on 19 July 2021 and closed on 23 February 2022. The online questionnaire was designed using Google Forms.

### 2.2. Questionnaire Structure and Data Cleaning

Questions 1–8 gathered demographic information about the veterinarians, including age, gender, current marital status, country of birth, population size of their residence, country of work, population size of their workplace and their field of specialization. Questions 9–13 focused on the respondents’ work details, such as their current job position, years of professional experience, average weekly work hours, the number of colleagues they work with, and the average length of annual leave.

The final section of the questionnaire used a 5-point Likert scale to assess how strongly veterinarians agreed or disagreed with various statements (where 1 = strongly disagree, 2 = disagree, 3 = undecided, 4 = agree, and 5 = strongly agree). This part of the survey examined 16 common stressors and strain-related factors experienced by veterinarians in their daily work and personal lives. These items were assessed in relation to their perceived impact on veterinary work and personal life and were thematically adapted from previously validated instruments addressing workload, client interactions and psychological strain [20,26,37,38]. The items were adapted to the aims of the present exploratory survey and were analyzed individually. The responses received were analyzed using Microsoft Excel^®^. Before analysis, the data were cleaned by checking for incomplete or logically inconsistent responses (e.g., selecting “more than 40 h of work” but also stating “I do not work currently”). As the questionnaire was fully anonymous, individual identification was not possible. However, all responses were manually reviewed for logical consistency and completeness to identify potential duplicates, and no duplicates were detected based on demographic and employment patterns.

### 2.3. Statistical Analysis

The data were analyzed using IBM SPSS Statistics Version 25 (IBM, Armonk, NY, USA) [39]. The individual veterinarian was the unit of analysis. The perceived impacts of work-related stressors and strain-related factors on veterinary work and personal life were assessed using a 5-point Likert scale (1 = strongly disagree, 2 = disagree, 3 = neither agree nor disagree, 4 = agree, 5 = strongly agree). Although Likert-scale responses are ordinal by design, mean scores (M) and standard deviations (SD) were calculated for each item to summarize response patterns and to allow comparison between groups. Before performing parametric tests, the distribution of the data was visually examined using histograms and Q-Q plots. Minor deviations from normality and variance equality were considered acceptable due to the sample size. As the Likert-based item scores showed an approximately normal distribution, they were treated as approximately continuous variables for parametric comparisons, and parametric methods (including independent-samples t-tests and one-way ANOVA) were applied where appropriate.

To examine whether veterinarians from different countries (Hungary, Finland, Sweden and Germany, *n* = 688) perceived the impact of 16 work-related stressors differently, one-way ANOVA was used to compare mean Likert-scale scores between countries. Data from other Northern European countries (i.e., Denmark, Estonia, Norway) were excluded from the inferential country-level analysis due to the small sample size (*n* = 36) and the heterogeneity of the responses across these countries, which made meaningful cross-national comparisons statistically unreliable. For each variable, the mean, standard deviation, *F*-value, degrees of freedom (*df*), *p*-value and 95% confidence interval (CI) were calculated.

Pearson’s chi-square (χ^2^) tests were used as supplementary analyses to examine differences in the distribution of Likert response categories across countries. The assumptions of the χ^2^ test, including expected cell counts, were checked before interpretation, and no substantial violations were found. Thus, ANOVA was used to compare mean scores, while χ^2^ tests were used to assess categorical response distributions. Chi-square results are reported with the χ^2^ value, *df*, *p*-value, and Cramer’s *V*. Cramer’s *V* was used as the effect size for χ^2^ tests. Values below 0.10 were interpreted as weak associations, values between 0.10 and 0.30 as small-to-moderate associations, and values above 0.30 as moderate or stronger associations. These thresholds were interpreted cautiously due to the exploratory design and the use of convenience samples.

The analyses of sociodemographic factors were conducted exclusively on the Hungarian sample (*n* = 236), as only this group included a sufficient number of respondents within each category to ensure the validity of statistical comparisons. All demographic variables (e.g., age group, gender, working hours, job position, holiday duration) were treated as grouping variables. Differences between age groups were assessed using one-way ANOVA, with means, standard deviations, *F*-values, *p*-values, and 95% CIs calculated for each stressor. Gender differences were examined using independent samples t-tests, preceded by Levene’s test for homogeneity of variances. The results include *t*-values, *df*, *p*-values, and 95% CI. Weekly working hours, job position, and holiday duration were analyzed using Pearson’s chi-square (χ^2^) tests, with Cramer’s *V* values reported to indicate the strength of associations.

To account for multiple comparisons, Bonferroni correction was applied separately within predefined families of comparisons. In the country-level comparisons, each family consisted of the 16 individual work-related and strain-related items within a given outcome domain, namely veterinary work and personal life. The same threshold was also applied in the demographic analyses within the Hungarian sample when the 16 items were tested across a given demographic or occupational factor. Therefore, the adjusted significance threshold was set at *p* ≤ 0.0031 (0.05/16) to control for Type I error. Results that did not meet this Bonferroni-adjusted threshold were interpreted as descriptive trends rather than statistically significant findings.

## 3. Results

### 3.1. Socio-Demographic Characteristics of the Respondents

Overall, 724 veterinarians fully completed the online questionnaire, and their responses were analyzed. We excluded 26 partially completed responses from the analysis. The majority of respondents worked in Hungary (32.6%) and Finland (30.1%), followed by Sweden (21.7%) and Germany (10.6%) and then other Northern European countries (5.0%), including Estonia, Denmark, and Norway. The respondents’ major socio-demographic characteristics and veterinary fields are summarized in Table 1 and Table 2.

### 3.2. Perceived Impact of Work-Related Stressors and Strain-Related Factors on Veterinary Work

**Figure 1:**
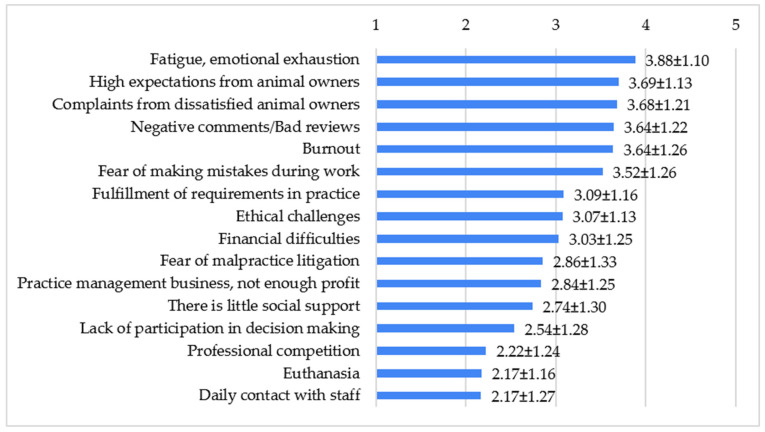
The veterinarians’ perception of stressors and strain-related factors and their perceived effects on veterinary work in the surveyed countries (mean values ± SD; *n* = 724). Note: Responses were given on a 5-point Likert scale, where 1 = strongly disagree, 2 = disagree, 3 = neither agree nor disagree, 4 = agree, 5 = strongly agree.

Fatigue, emotional exhaustion, high expectations from animal owners, complaints from dissatisfied animal owners, negative comments/bad reviews and burnout had the greatest impact on the veterinary work of all the responding veterinarians, all scoring above 3.5 points on a 5-point Likert scale. In contrast, professional competition, euthanasia and daily contact with staff were rated as the least impactful stressors, receiving the lowest mean scores (Figure 1).

Supplementary Table S1 presents the descriptive mean scores and standard deviations for each stressor and strain-related factor by country. The corresponding country-level statistical comparisons for the perceived impact of stressors and strain-related factors on veterinary work are shown in Table 3.

The results showed significant differences between countries for several stressors. These include euthanasia (ANOVA: *p* < 0.0001), fatigue, emotional exhaustion (ANOVA: *p* < 0.0001), fear of malpractice litigation (ANOVA: *p* < 0.0001), financial difficulties (ANOVA: *p* < 0.0031) and fulfilment of requirements in practice (ANOVA: *p* < 0.0031). Veterinarians working in different countries do not experience these workplace stressors in a uniform way, with some factors more strongly influencing work in one country and less in another. Veterinary respondents reported similar perceptions regarding the impact of certain stressors, such as burnout or lack of peer support, indicating a broadly similar pattern across the studied countries.

All countries had high average scores for fatigue and emotional exhaustion. Hungarian respondents perceived this factor as having the most significant impact on their work (mean: 4.15 ± 1.05). The lowest scores were observed in Sweden (mean: 3.77 ± 1.14) and in Finland (mean: 3.68 ± 1.06) (ANOVA: *p* < 0.0001). Ethical challenges were the most stressful among respondents from the other Northern European countries (mean: 3.39 ± 1.02) and Germany (mean: 3.18 ± 1.24), while they were much less likely to feel stressful among Finnish respondents (mean: 2.93 ± 1.05) (χ^2^ test: *p* < *0.001*). Fear of malpractice litigation was least prevalent among German (mean: 2.72 ± 1.20) and Hungarian (mean: 2.60 ± 1.29) respondents, while this stressor was much more prominent among Swedish respondents (mean: 3.24 ± 1.26) (ANOVA: *p* < 0.0001). Regarding the issue of being excluded from decision-making, Hungarian respondents perceived this as the least problematic factor (mean: 2.42 ± 1.38) among all countries, followed by respondents in Sweden (mean: 2.66 ± 1.30) and those in the other Northern European countries (mean: 2.91 ± 1.15). Professional competition had the greatest perceived impact among Hungarians (mean: 2.38 ± 1.30) and the smallest among Swedish respondents (mean: 2.04 ± 1.26). There were particularly large differences in perceptions of euthanasia: the majority of Finnish (mean: 1.68 ± 0.83) and Swedish veterinarians (mean: 1.88 ± 0.95) did not perceive it as a source of stress, whereas German (mean: 2.41 ± 1.17) and Hungarian (mean: 2.64 ± 1.27) respondents reported feeling its impact more strongly (ANOVA: *p* < 0.0001).

### 3.3. Perceived Impact of Work-Related Stressors and Strain-Related Factors on Personal Life

**Figure 2:**
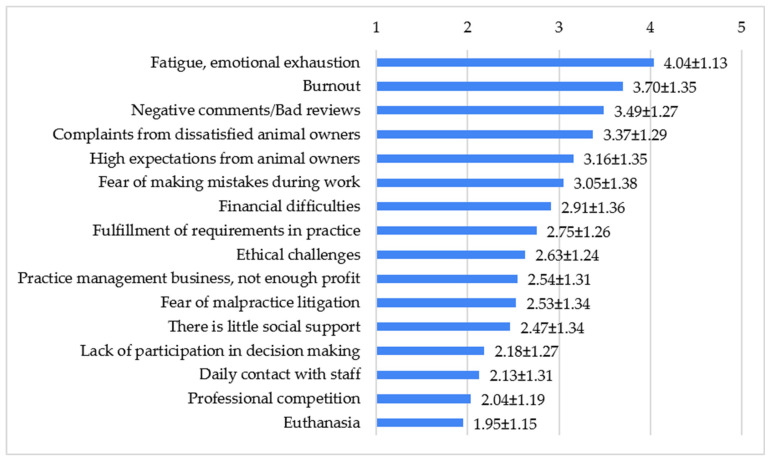
The veterinarians’ perception of stressors and strain-related factors and their effects on their personal life in the surveyed countries (mean values ± SD; *n* = 724). Note: Responses were given on a 5-point Likert scale, where 1 = strongly disagree, 2 = disagree, 3 = neither agree nor disagree, 4 = agree, 5 = strongly agree.

Fatigue, emotional exhaustion, burnout and negative comments/bad reviews had the highest impacts on the responding veterinarians’ personal lives, with scores above 3.5 points. In contrast, daily contact with staff, professional competition and euthanasia were rated as the least impactful stressors (Figure 2).

Table 4 shows the country-level statistical comparisons of the perceived impact of work-related stressors and strain-related factors on personal life.

Significant differences were observed for certain factors, suggesting that veterinarians perceived the impact of these stressors on their private lives differently across countries. The most significant differences were for euthanasia (ANOVA: *p* < 0.0001), fatigue, emotional exhaustion (ANOVA: *p* < 0.0001), complaints from dissatisfied animal owners (ANOVA: *p* < 0.001) and fear of malpractice litigation (ANOVA: *p* < 0.001). Veterinary respondents reported similar perceptions regarding the impact of certain stressors, such as burnout, financial difficulties, professional competition or lack of participation in decision-making, indicating a broadly similar pattern across the studied countries.

Fatigue and emotional exhaustion scored high in all countries surveyed, but the strongest effects were reported by respondents from Hungary (mean: 4.17 ± 1.30) and from the other Northern European countries (mean: 4.22 ± 1.10). Slightly smaller values were found among Swedish (mean: 4.01 ± 1.15) and Finnish veterinarians (mean: 3.94 ± 1.01), and uncertainty was most pronounced among German respondents (mean: 3.86 ± 1.23) (ANOVA: *p* < 0.0001). The comments of complaining pet owners had the most negative impact on the well-being of Swedish respondents (mean: 3.73 ± 1.15), while a smaller proportion of Hungarian respondents perceived this as a problem (mean: 3.20 ± 1.35). Fear of making mistakes during work was the least common stressor among Finnish (mean: 2.82 ± 1.36) and Hungarian (mean: 3.06 ± 1.46) veterinarians, while it was more common among Swedish (mean: 3.26 ± 1.31) respondents. The burden of fulfilling professional requirements was particularly divisive, with many Finnish (mean: 2.60 ± 1.25) and Swedish (mean: 2.58 ± 1.14) respondents disagreeing that it linked to their personal life. Among Hungarian respondents, this stressor was significantly more often perceived as a major source of discomfort (mean: 2.97 ± 1.36) (χ^2^ test: *p* < 0.0031). Daily contact with colleagues was perceived as having no impact on personal life by the respondents in Sweden (mean: 1.89 ± 1.21) and Finland (mean: 1.91 ± 1.89), compared to Hungarian veterinarians (mean: 2.43 ± 1.42). There were significant differences on the issue of euthanasia. Many Hungarian (mean: 2.38 ± 1.30) veterinarians thought that it had an impact on their personal life, suggesting that euthanasia is a more emotionally distressing factor for them, compared to Swedish (mean: 1.76 ± 0.98) and Finnish (mean: 1.44 ± 0.81) respondents (ANOVA: *p* < 0.0001).

### 3.4. Differences in the Perceived Impact of Stressors and Strain-Related Factors by Demographic and Workplace Factors Among Hungarian Veterinarians

The analysis explores how various stressors impact veterinary work and personal life, considering gender (*n* = 235) (using T-Test), as well as age group (*n* = 236) (using One-Way ANOVA), weekly working hours (*n* = 231), job position (*n* = 220), and the number of annual holidays (*n* = 236) (using Chi-square tests) (Supplementary Tables S2–S6). Significant age-related differences were observed, with younger veterinarians (aged 23–34) consistently reporting higher levels of work- and life-related stress, particularly in relation to burnout (mean: 3.86 ± 1.26) (ANOVA: *p* < 0.0001), emotional exhaustion (mean: 4.36 ± 0.97) (ANOVA: *p* < 0.0001), client-related challenges (mean: 3.83 ± 1.11) (ANOVA: *p* < 0.0031) and fear of making mistakes during work (mean: 3.99 ± 1.13) (ANOVA: *p* < 0.0001) compared to their older counterparts (>54 years). Based on gender differences, female veterinary respondents consistently reported higher stress levels across multiple stressors, particularly in relation to burnout (mean: 3.80 ± 1.23) (T-test: *p* < 0.001), emotional exhaustion (mean: 4.35 ± 1.03) (*t*-test: *p* < 0.0001), ethical challenges (mean: 2.84 ± 1.22) (T-test: *p* < 0.0001), fear of making mistakes during work (mean: 3.29 ± 1.42) (T-test: *p* < 0.0001) and negative comments and bad reviews (mean: 3.74 ± 1.21) (T-test: *p* < 0.0001) than male veterinary respondents. Moreover, significant associations were found for lack of participation in decision making, with those working more than 40 h per week generally reporting higher stress levels (mean: 2.25 ± 1.22) compared to those working less than 40 h per week (mean: 2.06 ± 1.31) (χ^2^ test: *p* < 0.0031). Several stressors showed significant associations with job position, with employees consistently reporting higher stress levels, particularly regarding fear of making mistakes (mean: 3.95 ± 1.15) (χ^2^ test: *p* < 0.0001) and lack of participation in decision making (mean: 2.79 ± 1.37) (χ^2^ test: *p* < 0.0001) than veterinary leaders. Respondents who took less than 14 paid holiday days per year showed higher levels of burnout (mean: 4.09 ± 1.30) and higher scores related to fear of making mistakes (mean: 3.90 ± 1.31), negative comments and bad reviews (mean: 3.90 ± 1.32), high expectations from clients (mean: 3.45 ± 1.46), and lack of social support (mean: 2.98 ± 1.50) compared to those who had more than 14 holidays per year.

### 3.5. Workplace Measures to Maintain Mental Health

**Figure 3:**
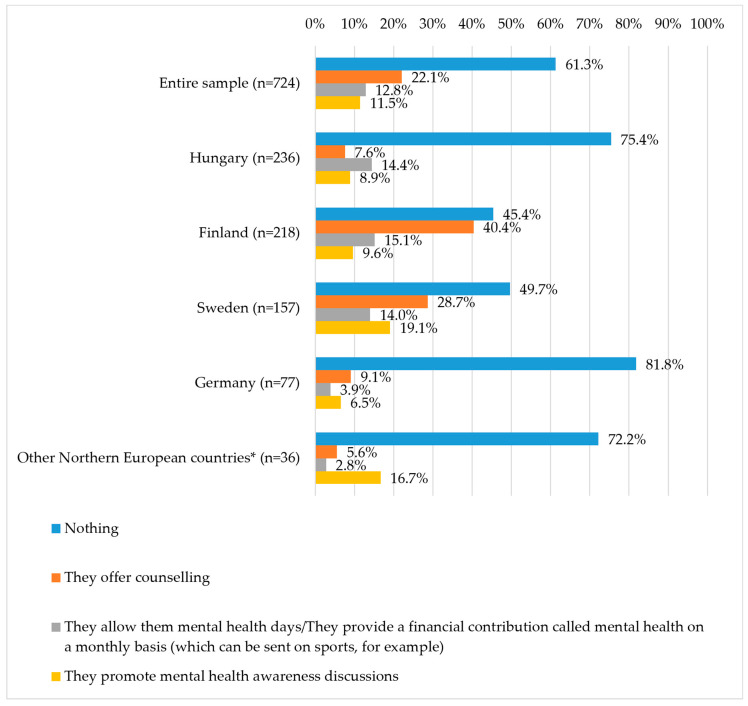
Workplace measures related to veterinarians’ mental health in the survey countries (*n* = 724). * Other Northern European countries include responses from veterinarians in Estonia, Denmark and Norway.

In the entire sample, nearly two-thirds (61.3%) of the responding veterinarians stated that their workplace did not contribute to maintaining a healthy mental state (Figure 3), with the highest proportions in Germany (81.8%) and Hungary (75.4%). The highest rates of offering workplace counselling were found in Finland (40.4%) and Sweden (28.7%). Overall, 12.8% of respondents reported that their workplace provided mental health days or financial contributions for well-being-related activities. This item referred to workplace mental health support measures and should be distinguished from annual holiday/free-day duration. The latter was preceded by the workplace promoting discussions on mental health awareness in Sweden (19.1%) and other Northern European countries (16.7%).

## 4. Discussion

This exploratory study examined veterinarians’ perceptions of work-related stressors and their perceived effects on professional and personal life in selected European samples. Overall, the findings suggest that fatigue, emotional exhaustion, burnout-related symptoms, client-related stressors, euthanasia-related stress, and workplace support are important themes in the surveyed respondents’ experiences.

### 4.1. Work-Related Stress and Its Consequences

Our findings suggest that occupational stress and strain-related symptoms remain relevant concerns among practicing veterinarians. Previous evidence has also linked work-related stressors with poorer mental health outcomes in the veterinary profession [40]. Beyond severe outcomes such as suicidal ideation, stress-related experiences may be associated with day-to-day difficulties in veterinary practice, including reduced concentration, impaired clinical judgement, challenges in client communication, and increased risk of medical errors [1,2,27,28,29]. Early-career veterinarians may be particularly vulnerable during the transition to practice, when sustained stress and burnout-related symptoms have been reported [41,42]. These factors have also been discussed in relation to suicide risk [26,43] and substance use problems [44]. Previous surveys have reported that many veterinarians perceive the profession as highly stressful, and this has been associated with distress, burnout-related symptoms, emotional exhaustion, and fatigue [36,43,45,46,47,48].

In the present study, respondents in the Hungarian sample reported the highest mean scores for stress and emotional exhaustion, whereas respondents in the Finnish and Swedish samples reported the lowest scores. These differences should be interpreted cautiously, as the samples were not nationally representative and recruitment methods differed between countries. Nevertheless, the observed patterns may be considered in relation to possible differences in job demands and organizational resources, including perceived workplace support for mental health, recovery-supportive practices, and collegial support [36,49,50]. In our survey, Finnish and Swedish respondents more frequently reported workplace-based support measures, such as counselling services and designated mental health days. These findings suggest that organizational-level support may be an important area for further research and workplace intervention. Previous studies have also associated poorer well-being among veterinarians with work-related stressors such as overwork, lack of professional appreciation, workplace conflicts, and ethical dilemmas [3,4,18,20,29,51,52]. The increased suicide risk reported in the veterinary profession remains an important warning sign and underlines the need for preventive strategies and accessible mental health support [18,20,53].

### 4.2. Stress Related to Client Interactions and Euthanasia

Although veterinary work inherently involves client interactions, euthanasia was examined separately because it represents a recurrent and emotionally charged end-of-life situation that may combine client-related stress with moral and ethical strain [15,16,17,48,54]. Our findings suggest that client-related stressors and ethical challenges are interrelated in veterinary work. High client expectations, complaints, and negative comments or reviews were among the most relevant external stressors reported by respondents. This is consistent with previous studies showing that veterinarians may experience pressure from client interactions, workplace demands, conflicts with clients or colleagues, and poor work–life balance [18,20,29,46,55]. In our data, negative comments or reviews were also related to respondents’ personal well-being, suggesting that managing dissatisfied clients may represent an important perceived burden. These findings support the need for communication and conflict-management training in veterinary practice [55].

Ethical challenges may include balancing animal welfare, client expectations, financial limitations, and end-of-life decisions [15]. Euthanasia is a key example, particularly when owners have strong emotional attachments or conflicting views [16,17]. In the present study, euthanasia was rated as a lower-intensity stressor in Finland and Sweden than in Hungary and Germany, although interpretation should remain cautious because the samples were not nationally representative. These differences may reflect variation in professional attitudes, end-of-life decision-making practices, ethical frameworks, communication norms, and workplace support systems [56]. Previous studies have also reported that euthanasia may be associated with stress among veterinarians [54,57], although it may not always be experienced as a major stressor unless the veterinarian has formed an attachment to the animal or identifies strongly with the owner’s experience [55].

In the Hungarian sample, female respondents reported higher perceived burden related to ethical challenges. This finding may be interpreted in relation to previous literature suggesting that women in veterinary medicine often report higher emotional involvement and empathy [58]. However, gendered expectations, professional roles, and emotional responses to ethical burden were not directly measured in the present study and should therefore be considered possible contextual explanations rather than conclusions demonstrated by our data [59,60]. Previous international findings also reported higher ethical burden in Hungary than in Sweden, and higher euthanasia-related ethical burden among women [18]. Evidence from Slovakia further suggests that coping strategies such as emotional regulation, rationalization, and peer support may be relevant when managing ethically difficult situations [61].

### 4.3. Age and Gender-Specific Experiences of Stress and Burnout in Hungary

In the Hungarian sample, younger respondents aged 23–34 years reported higher scores for several work-related stressors and strain-related symptoms, including fatigue, emotional exhaustion, burnout-related symptoms, complaints from animal owners, and fear of making mistakes. These patterns may reflect the challenges of early career stages, when veterinarians are still developing coping strategies and adjusting their expectations to the realities of practice [36,62]. Previous literature suggests that limited or uneven career guidance may contribute to misaligned expectations and early dissatisfaction in veterinary medicine [63,64]. Similar age-related patterns have also been reported among Hungarian medical doctors, where age was negatively correlated with emotional exhaustion and depersonalization but positively correlated with personal efficacy [65].

Gender-related differences in the Hungarian sample should also be interpreted cautiously. Female respondents reported higher scores for several stressor-related items, including fear of making mistakes, high expectations from animal owners, emotional exhaustion, burnout-related symptoms, and negative comments or reviews. This is consistent with previous veterinary studies showing that emotional exhaustion and stress-related symptoms are more frequently reported among female veterinarians [26,28,36,48,62]. By contrast, male respondents’ concerns were more often related to earnings, similar to findings from New Zealand [62]. These patterns should also be considered in the context of the strong feminization of the veterinary profession in Hungary, where the proportion of female veterinarians is steadily increasing and now predominant [66,67]. Previous research also suggests that emotional exhaustion is related not only to gender but also to work environment characteristics, emotional demands, and perceived organizational support [68].

Possible explanations for these gender-related patterns include workplace expectations, emotional labor, work–family conflict, sociocultural expectations, and coping styles; however, these mechanisms were not directly measured in the present study and should therefore be considered contextual interpretations rather than conclusions demonstrated by our data. Previous research suggests that women working in historically male-dominated professional contexts may face higher performance and credibility expectations [59,69]. Emotional labor and the need to manage client relationships may also be relevant in veterinary practice, particularly where professional and family responsibilities overlap [70,71]. Differences in coping styles have also been proposed as one possible explanation for gender differences in perceived stress and emotional exhaustion [72,73].

Work–life balance may be another relevant contextual factor. Previous studies have reported that female veterinarians and physicians may experience more difficulty in setting boundaries between their professional and personal lives, particularly in professions characterized by long working hours, emotional demands, and irregular schedules [70,74,75]. In Hungarian physicians, work–family conflict was associated with burnout dimensions, especially emotional exhaustion and depersonalization, and this relationship may be shaped by traditional gender roles and cultural expectations [75]. Another Hungarian study found higher depersonalization among male physicians, while caregiving burden was associated with fatigue and depersonalization among women [65]. Taken together, our findings and the previous literature suggest that workplace-level resources, including workload management, team support, supportive leadership, and access to mental health resources, may be important for reducing emotional exhaustion and burnout-related symptoms among veterinarians [76].

### 4.4. Perceived Impact of Job Position, Working Hours, and Holiday Duration in Hungary

In the Hungarian sample, respondents in non-managerial positions reported higher scores for several stressor-related items than owners or managers, particularly fear of making mistakes, concerns about malpractice claims and litigation, limited involvement in decision-making, and negative feedback. This differs from some studies from the United States and Europe, where practice owners reported higher stress levels [36,48]. In our sample, the higher scores among non-managerial veterinarians may be interpreted in relation to lower job control, reduced decision autonomy, and limited influence over work organization [68]. These factors may be associated with higher emotional demands when combined with professional responsibility and client-facing work. Previous studies also suggest that such stressor-related experiences may be linked to difficulties in clinical performance, decision-making confidence, and client communication [1,2,9,28]. Respondents working more than 40 h per week also reported higher perceived burden related to interactions with colleagues, social support, and involvement in workplace decision-making [18,20,55].

Fear of professional mistakes was particularly common among younger, female, and non-managerial respondents in Hungary, which is consistent with previous findings from England [18,20]. These patterns may partly reflect differences in practice models, workplace support systems, and opportunities for professional development. Previous studies have highlighted the possible role of peer support, paired field inspections, and continuing professional development frameworks in reducing stress and fatigue and maintaining veterinary competencies [49,77].

Holiday duration showed mainly descriptive patterns. Respondents who took fewer than 14 consecutive days of annual paid leave reported higher scores for several stressor-related items, particularly in relation to personal life. Jansen et al. reported that only 9% of veterinarians in Hungary took at least two weeks of leave due to mental health problems, including burnout, exhaustion, compassion fatigue, and depression, which was among the lowest rates in Europe [36]. Burnout, fear of making mistakes, and negative comments also showed descriptive associations with holiday duration [78]. However, these findings should be interpreted as non-significant descriptive trends rather than confirmed associations.

### 4.5. Workplace Culture and Recovery

Mental health challenges among veterinarians may be associated with reduced help-seeking behavior [79]. Although some veterinarians develop personal coping strategies, recognizing stress-related difficulties remains important for both professional functioning and personal well-being [4]. Darby et al. [80] found that lack of time for self-care was a major barrier to healthy coping, while approach-focused coping strategies were positively associated with resilience.

Our findings suggest that many respondents perceived limited workplace-level support for veterinarians’ mental health. Recovery may require time away from work, rest, and activities outside professional duties. Strategies such as clearer boundaries between work and personal life, mentoring programs, and counselling may support post-work recovery [62,81]. In our survey, workplace-level mental health measures differed between country-level samples. More than two-thirds of respondents reported that their workplace did not actively support mental well-being, with particularly high proportions in Germany and Hungary, whereas Finnish and Swedish respondents more frequently reported access to counselling services and mental health awareness initiatives [36,40,50].

Workplace culture and management practices may shape how veterinarians experience work-related stress, particularly through leadership style, decision autonomy, perceived organizational support, and recovery opportunities [68,76]. Organizational resources and supportive management have been described as protective factors against burnout-related symptoms and secondary traumatic stress while also supporting compassion satisfaction and sustainable professional functioning [76]. These findings suggest that workplace-level support, rather than individual coping alone, should be considered an important target for future interventions. Practices such as proactive managerial involvement, accessible counselling services, and visible mental health policies may provide useful examples for improving veterinary workplace well-being, although direct cross-country conclusions should be drawn cautiously [36,50,68,76].

### 4.6. Limitations

Several methodological limitations should be considered. First, participants were recruited through non-probability convenience sampling via online channels, professional networks, mailing lists, and social media groups. Therefore, the samples cannot be considered nationally representative. Response rates could not be calculated, country-level sample sizes were unequal, and recruitment strategies differed between countries. Consequently, differences between country-level samples may partly reflect recruitment strategy, sample composition, professional specialization, age and gender structure, or workplace type rather than genuine national differences alone. The results should therefore be interpreted as exploratory and descriptive rather than as representative cross-country comparisons. Self-selection bias is also possible, as veterinarians with stronger experiences or opinions related to stress, mental health, client communication, or work–life balance may have been more likely to participate. The relatively high proportion of female and younger respondents may further limit the generalizability of age- and gender-specific findings [36,66,69].

Second, the data were based on self-reported Likert-type responses and may therefore be affected by recall bias, social desirability bias, and individual differences in response style. Although the questionnaire was developed based on the literature and pilot-tested for face validity, clarity, relevance, and comprehensibility, it was not formally psychometrically validated. The study did not use validated composite measures of burnout, stress, mental health, or work–life balance, but relied on individual self-reported items assessing perceived stressor-related effects. No formal cross-cultural validation was performed, and differences in language, cultural context, professional norms, or interpretation of scale anchors may have influenced how respondents understood and answered the items.

Third, although Bonferroni correction was applied to reduce the risk of Type I error, the large number of comparisons increases the possibility of both false-positive and false-negative findings. Results that did not meet the Bonferroni-adjusted significance threshold should therefore be interpreted only as descriptive trends. In addition, the analyses were primarily univariable and exploratory, and residual confounding by factors such as type of practice, workload intensity, income, workplace culture, availability of support, previous mental health history, or country-specific professional structures cannot be excluded.

Finally, the cross-sectional design prevents conclusions about temporal order or causality. The findings describe associations and perceived effects at one point in time but cannot establish whether specific work-related stressors caused poorer well-being, personal-life difficulties, or lower perceived workplace support. Longitudinal studies following veterinarians over time would be valuable to examine changes in professional functioning, personal life, and well-being and to clarify temporal relationships. Overall, the results should be interpreted as exploratory and descriptive findings from selected convenience samples rather than as causal, representative, or generalizable estimates for all veterinarians in the participating countries.

## 5. Conclusions

This exploratory study highlights that fatigue, emotional exhaustion, burnout-related symptoms, client-related stressors, and euthanasia-related stress were perceived as important burdens affecting veterinarians’ professional and personal lives in the surveyed European samples. In the Hungarian sample, younger and female veterinarians reported a greater perceived impact of several work-related stressors, indicating that targeted workplace support may be particularly important for these groups. The findings underline that stress reduction in veterinary practice should not rely solely on individual coping strategies. Organizational-level measures, including manageable workloads, adequate staffing, supportive leadership, clear role expectations, effective team communication, and access to mental health resources, may help reduce work-related strain and promote workplace well-being.

Despite its limitations, this study provides descriptive data from several Central and Northern European veterinary samples, including Hungary, Finland, Sweden, Germany, Denmark, Estonia, and Norway. To the best of our knowledge, few similarly broad surveys have examined work-related stressors and their perceived effects on veterinary work and personal life across these European samples. Therefore, the findings may serve as useful reference points for future research and workplace-level interventions, particularly regarding organizational support, communication practices, work–life balance, and mental health resources. Given the exploratory design, convenience sampling, and cross-sectional nature of the study, the findings should be interpreted as descriptive rather than representative or causal. Nevertheless, they identify key areas that veterinary workplaces, professional organizations, and educators can address in future support, prevention, and intervention strategies.

## Figures and Tables

**Table 1:** Socio-demographic characteristics of the respondents working in Hungary, Finland, Sweden, Germany and other Northern European countries (%).

Variable	Category	Total (*n* = 724)	Hungary (*n* = 236)	Finland (*n* = 218)	Sweden (*n* = 157)	Germany (*n* = 77)	Other Northern European Countries * (*n* = 36)
Age group	23–34 years	45.4%	47.9%	40.4%	41.4%	53.2%	61.1%
	35–54 years	46.4%	40.3%	51.4%	51.0%	45.5%	38.9%
	Over 54 years	8.1%	11.9%	8.3%	7.6%	1.3%	0.0%
Gender	Male	11.0%	23.4%	3.7%	5.1%	7.9%	5.6%
	Female	89.0%	76.6%	96.3%	94.9%	92.1%	94.4%
Working hours	Weekly < 40 h	49.7%	57.1%	53.2%	46.2%	33.3%	30.6%
	Weekly > 40 h	50.3%	42.9%	46.8%	53.8%	66.7%	69.4%
Position	Owner/manager position	28.8%	37.7%	28.9%	26.8%	14.7%	11.4%
	Employee/non-managerial position	71.2%	62.3%	71.1%	73.2%	85.3%	88.6%
Holidays	<14 days per year	9.7%	20.8%	5.0%	3.2%	5.2%	2.8%
	14–28 days per year	45.4%	55.9%	28.4%	48.4%	58.4%	38.9%
	>28 days per year	44.9%	23.3%	66.5%	48.4%	36.4%	58.3%

* Other Northern European countries include responses from veterinarians in Estonia, Denmark and Norway.

**Table 2:** Respondents’ veterinary field of work in Hungary, Finland, Sweden, Germany and other Northern European countries (%).

Veterinary Field of Work	Total (*n* = 724)	Hungary (*n* = 236)	Finland (*n* = 218)	Sweden (*n* = 157)	Germany (*n* = 77)	Other Northern European Countries * (*n* = 36)
Small Animal Medicine	69.6%	78.0%	66.5%	65.6%	57.1%	77.8%
Mixed Animal Practice	18.0%	12.3%	27.5%	18.5%	13.0%	5.6%
Equine Medicine	15.9%	6.4%	17.4%	25.5%	23.4%	11.1%
Farm Animal Medicine	13.4%	10.6%	11.9%	16.6%	15.6%	22.2%
Authorities/State Vet	10.8%	9.3%	16.1%	9.6%	6.5%	2.8%
Teaching/Research	9.3%	6.4%	9.6%	5.7%	15.6%	27.8%
Exotic Animal Medicine	9.0%	14.0%	6.9%	7.0%	5.2%	5.6%
Laboratory	3.0%	3.8%	0.9%	1.3%	6.5%	11.1%
Other	3.2%	4.2%	1.4%	3.2%	6.5%	0.0%

* Other Northern European countries include responses from veterinarians in Estonia, Denmark and Norway. “Other” category included professionals working in the pharmaceutical industry, the fish and rabbit sector, shelters, etc. As multiple responses were allowed, percentages may add up to more than 100%.

**Table 3:** Country-level differences between Hungary, Finland, Sweden and Germany in the perceived impact of stressors and strain-related factors on veterinary work (*n* = 688).

Stressor-Related Items (*n* = 16)	Effect on Veterinary Work							
	Pearson’s Chi-Square (χ^2^) Test			Cramer’s *V* Value	ANOVA			
	χ^2^	*df*	*p* Value		*F*	*df*_Between, *df*_Within Groups	*p* Value	95% CI
Burnout	10.377	12	*p* = 0.583	0.075	0.069	3, 617	*p* = 0.976	(3.51–3.71)
Complaints from dissatisfied animal owners	15.480	12	*p* = 0.216	0.089	3.973	3, 651	*p* = 0.008	(3.57–3.76)
Daily contact with staff	17.154	12	*p* = 0.144	0.096	3.024	3, 615	*p* = 0.029	(2.06–2.26)
Ethical challenges	37.926	12	*p* < 0.001 *	0.138	1.307	3, 662	*p* = 0.271	(2.97–3.14)
Euthanasia	99.546	12	*p* < 0.0001 *	0.231	32.356	3, 617	*p* < 0.0001 *	(2.05–2.24)
Fatigue, emotional exhaustion	47.220	12	*p* < 0.0001 *	0.153	8.208	3, 688	*p* < 0.0001 *	(3.78–3.95)
Fear of making mistakes during work	15.818	12	*p* = 0.200	0.089	1.863	3, 657	*p* = 0.134	(3.41–3.60)
Fear of malpractice litigation	43.366	12	*p* < 0.0001 *	0.151	7.403	3, 634	*p* < 0.0001 *	(2.74–2.94)
Financial difficulties	15.082	12	*p* = 0.237	0.088	4.652	3, 648	*p* < 0.0031 *	(2.91–3.11)
Fulfilment of requirements in practice	14.712	12	*p* = 0.258	0.086	2.974	3, 654	*p* < 0.0031 *	(3.00–3.18)
High expectations from animal owners	19.031	12	*p* = 0.088	0.098	1.473	3, 651	*p* = 0.221	(3.59–3.77)
Lack of participation in decision making	27.051	12	*p* = 0.008	0.120	1.023	3, 624	*p* = 0.382	(2.42–2.62)
Negative comments, bad reviews	14.090	12	*p* = 0.295	0.084	2.175	3, 654	*p* = 0.090	(3.57–3.75)
Practice management business, not enough profit	12.806	12	*p* = 0.383	0.085	1.134	3, 592	*p* = 0.335	(2.72–2.92)
Professional competition	28.161	12	*p* = 0.005	0.122	2.594	3, 622	*p* = 0.052	(2.12–2.31)
There is little social support	9.645	12	*p* = 0.647	0.071	0.535	3, 642	*p* = 0.658	(2.64–2.84)

Note: *p*-values with asterisk (*) indicate statistically significant differences (Bonferroni-adjusted *p* ≤ 0.0031) between countries in veterinarians’ perceptions of the impact of specific stressors.

**Table 4:** Country-level differences between Hungary, Finland, Sweden and Germany in the perceived impact of stressors and strain-related factors on personal life (*n* = 688).

Stressor-Related Items (*n* = 16)	Effect on Personal Life							
	Pearson’s Chi-Square (χ^2^) Test			Cramer’s *V* Value	ANOVA			
	χ^2^	*df*	*p* Value		*F*	*df*_Between, *df*_Within Groups	*p* Value	95% CI
Burnout	16.588	12	*p* = 0.166	0.094	1.030	3, 616	*p* = 0.379	(3.55–3.77)
Complaints from dissatisfied animal owners	26.273	12	*p* = 0.009	0.116	5.603	3, 645	*p* < 0.001 *	(3.26–3.46)
Daily contact with staff	25.393	12	*p* = 0.013	0.119	7.154	3, 594	*p* = 0.087	(2.01–2.22)
Ethical challenges	15.004	12	*p* = 0.241	0.089	1.932	3, 632	*p* = 0.123	(2.51–2.70)
Euthanasia	81.395	12	*p* < 0.0001 *	0.215	25.951	3, 582	*p* < 0.0001 *	(1.84–2.02)
Fatigue, emotional exhaustion	36.414	12	*p* < 0.0001 *	0.135	2.201	3, 665	*p* < 0.0001 *	(3.94–4.11)
Fear of making mistakes during work	21.161	12	*p* = 0.048	0.105	3.124	3, 640	*p* = 0.025	(2.93–3.14)
Fear of malpractice litigation	20.368	12	*p* = 0.060	0.105	5.596	3, 612	*p* < 0.001 *	(2.42–2.63)
Financial difficulties	12.263	12	*p* = 0.425	0.080	2.887	3, 639	*p* = 0.035	(2.77–2.98)
Fulfilment of requirements in practice	31.802	12	*p* < 0.0031 *	0.129	4.220	3, 632	*p* = 0.006	(2.66–2.85)
High expectations from animal owners	20.585	12	*p* = 0.057	0.103	3.739	3, 637	*p* = 0.011	(3.04–3.25)
Lack of participation in decision making	15.532	12	*p* = 0.214	0.093	1.359	3, 598	*p* = 0.254	(2.07–2.27)
Negative comments, bad reviews	13.124	12	*p =* 0.360	0.082	1.810	3, 646	*p* = 0.144	(3.39–3.59)
Practice management business, not enough profit	16.684	12	*p* = 0.162	0.098	3.136	3, 570	*p* = 0.025	(2.43–2.64)
Professional competition	12.027	12	*p* = 0.443	0.081	1.750	3, 603	*p* = 0.156	(1.93–2.12)
There is little social support	5.485	12	*p* = 0.940	0.054	0.727	3, 615	*p* = 0.536	(2.36–2.57)

Note: *p*-values with asterisk (*) indicate statistically significant differences (Bonferroni-adjusted *p* ≤ 0.0031) between countries in veterinarians’ perceptions of the impact of specific stressors.

## Data Availability

The original contributions presented in this study are included in the article/Supplementary Material. Further inquiries can be directed to the corresponding author.

## Supplementary Materials

The following supporting information can be downloaded at: https://www.mdpi.com/article/10.3390/vetsci13060583/s1, Supplementary Questionnaire; Table S1: Country-level comparison of respondents’ perceived impact of work-related stressors and strain-related factors on veterinary work and personal life (*n* = 724); Table S2: Age-group differences in the assessment of work-related stressors and strain-related factors among Hungarian veterinarians (*n* = 236); Table S3: Gender differences in the assessment of work-related stressors and strain-related factors among Hungarian veterinarians (*n* = 235); Table S4: Working-hour differences in the assessment of work-related stressors and strain-related factors among Hungarian veterinarians (*n* = 231); Table S5: Job-position differences in the assessment of work-related stressors and strain-related factors among Hungarian veterinarians (*n* = 220); Table S6: Holiday-duration differences in the assessment of work-related stressors and strain-related factors among Hungarian veterinarians (*n* = 236).
