# Supplementary material for: Work-Related Stressors and Their Perceived Impact on Veterinary Work and Personal Life: A Multi-Country European Study
Source: Vet Sci. 2026 Jun 15;13(6):583. doi: 10.3390/vetsci13060583 (PMC13308442; doi:10.3390/vetsci13060583)
Supplement: Supplementary file 1 [file vetsci-13-00583-s001.zip › vetsci-4370239-supplementary.pdf]

Supplementary Materials

Supplementary Questionnaire. Questionnaire description

This research is conducted on issues related to mental health in the veterinary profession and explores work-related and personal stressors affecting the mental health and well-being of veterinarians. The aim is to understand how various stress factors impact veterinarians' professional and private lives. The questionnaire is being distributed to veterinarians in several countries, and the responses will be compared across countries to provide a comprehensive overview of the challenges veterinarians face in different regions.

Participation in the study is voluntary and anonymous. By submitting the questionnaire, participants provide their written informed consent to take part in the research. No personal or identifiable information is collected, and all data will be used solely for academic purposes in accordance with data protection regulations. The data will be processed in aggregate form. The questionnaire takes approximately 15 minutes to complete.

Thank you for contributing to our growing understanding of mental health in the veterinary field!

Q 1: Which age group do you belong to?

- 23-34
- 35-44
- 45-54
- 55-64
- 65-74
- Older than 74

Q 2: What is your gender?

- Female
- Male
- I prefer not to say.

Q 3: What is your current marital status?

- Unmarried
- Married
- Divorced
- Widow
- Other: ___________________________

Q 4: Where is your place of residence?[1]

[1] For Hungarian Veterinarians:
- Capital city
- Large town (>100,000)

- <10,000
- 10,000-100,000
- 100,001-500,000
- 500,001-1,000,000
- >1,000,000

Q5: Please write the name of the country where you were born!

Q 6: Where is your place of work? (You may choose more than one answer!)[2]

- <10,000
- 10,000-100,000
- 100,001-500,000
- 500,001-1,000,000
- >1,000,000

Q 7: Please write the name of the country where you work!

Q 8: What field are you currently working in within veterinary medicine? (You may choose more than one answer!)

- Small Animal Medicine
- Exotic Animal Medicine
- Farm Animal Medicine
- Equine Medicine
- Mixed Practice
- Laboratory
- Authorities/State Vet
- Teaching/Research
- Other: ____________________

Q 9: What position are you in at your current job now?

- Owner / manager position
- Employee / non - managerial position
- Retired
- Other: _________________________

Q 10: How many years of professional experience do you have?

- Middle-sized town (50,001-100,000)
- Small town (10,000-50,000)
- Other settlement (<10,000)

[2] For Hungarian Veterinarians:

- Capital city
- Large town (>100,000)
- Middle-sized town (50,001-100,000)
- Small town (10,000-50,000)
- Other settlement (<10,000)

- Less than 1 year
- 1-3 years
- 4-6 years
- 7-10 years
- 11-20 years
- 21-30 years
- 30 <

Q 11: Which of the following categories most accurately describes your average weekly work hours?

- weekly less than 20 hours
- weekly 20-30 hours
- weekly 31-40 hours
- weekly 41-50 hours
- weekly 51-60 hours
- weekly over 60 hours
- Unemployed, looking for a job.
- Unemployed, not looking for a job.

Q 12: How many colleagues do you work with on a daily basis in your workplace?

- Alone
- 1-5 people
- 6-10 people
- 11-20 people
- More than 20 people.

Q 13: How much holiday/free days are you given a year?

- Less than 14 days
- 14-28 days
- 29-35 days
- 36-42 days
- More than 42 days

Q 14: Evaluate the following stressors related to veterinary work and how they affect your veterinary work:

- Burn-out
- Fatigue, emotional exhaustion
- Euthanasia
- Ethical challenges
- Financial difficulties
- Complaints from dissatisfied animal owners
- High expectations of animal owners
- Negative comments/bad reviews
- Fulfilment of requirements in practice
- Practice management business, not enough profit
- Fear of making mistakes in the profession
- Daily contact with staff

- Professional competition
- Fear of malpractice litigation
- There is little social support
- Lack of participation in decision - making

Q 15: Evaluate the following stressors related to veterinary work, and how they affect your mental health in your private life:

- Burn-out
- Fatigue, emotional exhaustion
- Euthanasia
- Ethical challenges
- Financial difficulties
- Complaints from dissatisfied animal owners
- High expectations of animal owners
- Negative comments/bad reviews
- Fulfilment of requirements in practice
- Practice management business, not enough profit
- Fear of making mistakes in the profession
- Daily contact with staff
- Professional competition
- Fear of malpractice litigation
- There is little social support
- Lack of participation in decision - making

Q 16: How does your work regard mental health? (You may choose more than one answer!)

- They allow us mental health days
- They do mental health awareness discussions
- They offer counselling
- Nothing

Q 17: If you have any additional comments regarding anything mentioned in the questionnaire, please add them below:

____

Supplementary Table S1. Country-level comparison of respondents' perceived impact of work-related stressors and strain-related factors on veterinary work and personal life (n = 724).

Stressor-related items (*n*=16)	Effect on veterinary work										Effect on personal life									
	Hungary (*n*=236)		Finland (*n*=218)		Sweden (*n*=157)		Germany (*n*=77)		Northern-European countries* (*n*=36)		Hungary (*n*=236)		Finland (*n*=218)		Sweden (*n*=157)		Germany (*n*=77)		Other Northern-European countries* (*n*=36)	
	M	SD	M	SD	M	SD	M	SD	M	SD	M	SD	M	SD	M	SD	M	SD	M	SD
Burnout	3.65	1.25	3.60	1.28	3.60	1.28	3.60	1.27	4.06	1.15	3.60	1.38	3.71	1.35	3.77	1.33	3.45	1.37	4.31	1.01
Complaints from dissatisfied animal owners	3.60	1.25	3.53	1.27	3.95	1.10	3.63	1.20	3.91	0.98	3.20	1.35	3.31	1.29	3.73	1.15	3.21	1.36	3.70	1.16
Daily contact with staff	2.36	1.33	2.07	1.19	2.03	1.28	2.01	1.22	2.31	1.20	2.43	1.42	1.91	1.19	1.89	1.21	2.03	1.24	2.39	1.34
Ethical challenges	3.09	1.24	2.93	1.05	3.11	1.01	3.18	1.24	3.39	1.02	2.62	1.26	2.48	1.25	2.63	1.16	2.88	1.22	3.03	1.34
Euthanasia	2.64	1.27	1.68	0.83	1.88	0.95	2.41	1.17	2.69	1.21	2.38	1.30	1.44	0.81	1.76	0.98	2.09	1.15	2.27	1.10
Fatigue, emotional exhaustion	4.15	1.05	3.68	1.06	3.77	1.14	3.73	1.13	4.14	1.07	4.17	1.17	3.94	1.01	4.01	1.15	3.86	1.23	4.22	1.10
Fear of making mistakes during work	3.59	1.32	3.37	1.27	3.63	1.15	3.38	1.34	3.72	1.09	3.06	1.46	2.82	1.36	3.26	1.31	3.10	1.26	3.34	1.28
Fear of malpractice litigation	2.60	1.29	2.85	1.28	3.24	1.26	2.72	1.20	3.16	1.32	2.31	1.31	2.44	1.32	2.86	1.35	2.74	1.36	2.69	1.23
Financial difficulties	3.16	1.24	2.75	1.25	3.13	1.24	3.04	1.24	3.43	1.14	3.06	1.38	2.84	1.33	2.65	1.31	2.85	1.34	3.60	1.42
Fulfilment of requirements in practice	3.23	1.19	3.12	1.14	2.93	1.13	2.89	1.08	3.06	1.20	2.97	1.36	2.60	1.25	2.58	1.14	2.85	1.11	2.74	1.26
High expectations from animal owners	3.72	1.20	3.56	1.12	3.80	1.00	3.64	1.26	3.97	0.95	3.12	1.39	2.95	1.33	3.44	1.21	3.14	1.45	3.47	1.37
Lack of participation in decision making	2.42	1.38	2.49	1.21	2.66	1.30	2.58	1.19	2.91	1.15	2.19	1.30	2.07	1.29	2.16	1.18	2.42	1.29	2.39	1.27
Negative comments, bad reviews	3.66	1.26	3.52	1.24	3.86	1.15	3.67	1.17	3.31	1.14	3.49	1.30	3.35	1.33	3.67	1.18	3.52	1.23	3.60	1.12
Practice management business, not enough profit	2.93	1.32	2.84	1.21	2.68	1.21	2.79	1.26	3.06	1.26	2.74	1.31	2.52	1.30	2.28	1.28	2.51	1.30	2.73	1.31
Professional competition	2.38	1.30	2.20	1.19	2.04	1.26	2.07	1.09	2.38	1.24	2.14	1.20	1.90	1.10	1.96	1.18	2.13	1.27	2.28	1.37
There is little social support	2.83	1.31	2.68	1.31	2.70	1.31	2.72	1.20	2.67	1.29	2.57	1.36	2.42	1.36	2.42	1.32	2.35	1.25	2.49	1.40

*Other Northern-European countries include responses from veterinarians in Estonia, Denmark and Norway

Responses were given on a 5-point Likert scale, where 1 = strongly disagree, 2 = disagree, 3 = neither agree nor disagree, 4 = agree, 5 = strongly agree.

Supplementary Table S2. Age-group differences in the assessment of work-related stressors and strain-related factors among Hungarian veterinarians (*n* = 236).

Stressor-related items (*n*=16)	Age group	Effect on veterinary work					Effect on personal life				
		M	SD	One-Way ANOVA		95% CI (23-34, 35-54, >54) (Lower, Upper)	M	SD	One-Way ANOVA		95% CI (23-34, 35-54, >54) (Lower, Upper)
				F	*p* value				*F*	*p* value	
Burnout	23-34 years	3.85	1.10	7.431	*p<0.001**	3.63, 4.07	3.86	1.26	12.430	*p<0.0001**	3.61, 4.11
	35-54 years	3.62	1.24			3.36, 3.88	3.64	1.29			3.37, 3.91
	Over 54 years	2.68	1.57			1.93, 3.44	2.32	1.62			1.60, 3.03
Complaints from dissatisfied animal owners	23-34 years	3.83	1.11	7.025	*p<0.0031**	3.62, 4.04	3.47	1.36	5.444	*p*=0.004	3.21, 3.72
	35-54 years	3.53	1.28			3.26, 3.80	3.04	1.26			2.78, 3.31
	Over 54 years	2.84	1.40			2.26, 3.42	2.62	1.36			2.07, 3.16
Daily contact with staff	23-34 years	2.36	1.34	4.943	*p*=0.008	2.10, 2.61	2.59	1.47	4.651	*p*=0.010	2.31, 2.86
	35-54 years	2.57	1.36			2.28, 2.85	2.45	1.40			2.15, 2.75
	Over 54 years	1.65	0.89			1.29, 2.01	1.63	1.01			1.20, 2.05
Ethical challenges	23-34 years	3.19	1.25	1.517	*p*=0.222	2.95, 3.42	2.82	1.29	3.560	*p*=0.011	2.58, 3.07
	35-54 years	3.07	1.21			2.81, 3.32	2.50	1.23			2.24, 2.76
	Over 54 years	2.71	1.27			2.17, 3.24	2.16	1.07			1.72, 2.60
Euthanasia	23-34 years	2.75	1.28	0.875	*p*=0.418	2.51, 3.00	2.54	1.41	1.385	*p*=0.253	2.26, 2.82
	35-54 years	2.54	1.23			2.29, 2.80	2.24	1.15			1.99, 2.48
	Over 54 years	2.48	1.38			1.88, 3.07	2.26	1.29			1.70, 2.82
Fatigue, emotional exhaustion	23-34 years	4.35	0.88	19.925	*p<0.0001**	4.19, 4.52	4.36	0.97	22.402	*p<0.0001**	4.18, 4.55
	35-54 years	4.22	0.92			4.03, 4.41	4.31	1.07			4.09, 4.53
	Over 54 years	3.00	1.47			2.39, 3.61	2.85	1.43			2.27, 3.43
Fear of making mistakes during work	23-34 years	3.99	1.13	12.855	*p<0.0001**	3.78, 4.20	3.41	1.40	6.687	*p<0.0031**	3.14, 3.67
	35-54 years	3.36	1.30			3.08, 3.63	2.80	1.45			2.50, 3.11
	Over 54 years	2.78	1.55			2.16, 3.39	2.48	1.45			1.88, 3.08
Fear of malpractice litigation	23-34 years	2.74	1.36	1.716	*p*=0.182	2.47, 3.00	2.34	1.32	1.231	*p*=0.294	2.08, 2.61
	35-54 years	2.56	1.44			2.26, 2.87	2.38	1.35			2.09, 2.67
	Over 54 years	2.19	1.33			1.66, 2.71	1.92	1.14			1.44, 2.40
Financial difficulties	23-34 years	3.28	1.27	3.161	*p*=0.044	3.04, 3.52	3.12	1.35	3.431	*p*=0.034	2.86, 3.38
	35-54 years	3.17	1.20			2.91, 3.42	3.17	1.43			2.88, 3.47
	Over 54 years	2.60	1.08			2.15, 3.05	2.38	1.17			1.88, 2.87
Fulfilment of requirements in practice	23-34 years	3.39	1.11	5.558	*p*=0.004	3.18, 3.60	3.14	1.37	2.678	*p*=0.071	2.89, 3.40
	35-54 years	3.24	1.14			3.00, 3.48	2.89	1.31			2.61, 3.16
	Over 54 years	2.56	1.45			1.98, 3.13	2.50	1.36			1.95, 3.05
	23-34 years	4.00	1.02	13.916	*p<0.0001**	3.81, 4.19	3.34	1.40	3.640	*p*=0.028	3.07, 3.60

High expectations from animal owners	35-54 years	3.67	1.21			3.42, 3.92	3.02	1.34			2.74, 3.30
	Over 54 years	2.68	1.31			2.14, 3.22	2.58	1.36			2.03, 3.13
Lack of participation in decision making	23-34 years	2.58	1.38	1.969	*p*=0.142	2.32, 2.85	2.28	1.35	1.953	*p*=0.144	2.02, 2.54
	35-54 years	2.33	1.34			2.05, 2.62	2.20	1.27			1.93, 2.48
	Over 54 years	2.00	1.48			1.34, 2.66	1.67	1.11			1.16, 2.17
Negative comments, bad reviews	23-34 years	3.88	1.14	7.300	*p<0.001**	3.66, 4.09	3.77	1.24	6.669	*p<0.0031**	3.53, 4.00
	35-54 years	3.60	1.25			3.34, 3.86	3.32	1.28			3.05, 3.58
	Over 54 years	2.83	1.49			2.20, 3.46	2.83	1.34			2.27, 3.40
Practice management business, not enough profit	23-34 years	2.72	1.37	3.898	*p*=0.022	2.42, 3.02	2.52	1.35	2.748	*p*=0.067	2.23, 2.82
	35-54 years	3.23	1.22			2.96, 3.50	2.99	1.24			2.71, 3.26
	Over 54 years	2.64	1.32			2.10, 3.18	2.63	1.28			2.08, 3.17
Professional competition	23-34 years	2.35	1.29	0.088	*p*=0.916	2.10, 2.59	2.16	1.15	0.192	*p*=0.826	1.94, 2.38
	35-54 years	2.42	1.32			2.15, 2.70	2.15	1.30			1.87, 2.43
	Over 54 years	2.41	1.37			1.87, 2.95	2.00	1.13			1.54, 2.46
There is little social support	23-34 years	3.08	1.31	5.554	*p*=0.004	2.82, 3.33	2.65	1.41	1.752	*p*=0.176	2.38, 2.92
	35-54 years	2.73	1.27			2.47, 2.99	2.60	1.34			2.32, 2.89
	Over 54 years	2.16	1.25			1.64, 2.68	2.08	1.14			1.60, 2.56

An asterisk (*) denotes a statistically significant association (Bonferroni-adjusted $p \leq 0.0031$).

Responses were given on a 5-point Likert scale, where 1 = strongly disagree, 2 = disagree, 3 = neither agree nor disagree, 4 = agree, 5 = strongly agree.

Supplementary Table S3. Gender differences in the assessment of work-related stressors and strain-related factors among Hungarian veterinarians (*n* = 235).

Stressor-related items (*n*=16)	Gender	Effect on veterinary work: M	SD	Levene's Test: *F*	Levene's Test: *p* value	Independent Samples T-Test: *t*	*df*	*p* value	95% CI (Upper, Lower)	Effect on personal life: M	SD	Levene's Test: *F*	Levene's Test: *p* value	T-Test: *t*	*df*	*p* value	95% CI (Upper, Lower)
Burnout	Male	3.17	1.42	3.971	*p*=0.047	2.617	63.230	*p*=0.004	-1.054, -0.141	2.98	1.66	16.925	*p<0.0001**	3.193	64.926	*p<0.001**	-1.334, -0.307
	Female	3.77	1.17							3.80	1.23						
Complaints from dissatisfied animal owners	Male	3.11	1.31	1.546	*p*=0.215	3.121	80.357	*p<0.0031**	-1.031, -0.228	2.46	1.24	1.579	*p*=0.210	4.803	87.347	*p<0.0001**	-1.350, -0.560
	Female	3.74	1.19							3.42	1.30						
Daily contact with staff	Male	2.09	1.23	2.755	*p*=0.098	1.805	93.600	*p*=0.074	-0.749, 0.036	2.04	1.21	5.958	*p*=0.015	2.490	94.791	*p*=0.026	-0.916, -0.103
	Female	2.45	1.35							2.55	1.46						
Ethical challenges	Male	2.63	1.33	2.522	*p*=0.114	2.849	74.910	*p<0.0031**	-0.999, -0.177	1.96	1.14	1.906	*p*=0.169	4.759	90.948	*p<0.0001**	-1.241, -0.510
	Female	3.22	1.18							2.84	1.22						
Euthanasia	Male	2.20	1.37	2.099	*p*=0.149	2.697	75.351	*p*=0.004	-1.002, -0.151	1.92	1.20	1.359	*p*=0.245	3.171	92.676	*p*=0.002	-1.009, -0.232
	Female	2.77	1.21							2.54	1.30						
Fatigue, emotional exhaustion	Male	3.62	1.25	9.029	*p<0.0031**	3.666	68.469	*p<0.0001**	-1.062, -0.314	3.57	1.39	16.881	*p<0.0001**	3.778	69.967	*p<0.0001**	-1.193, -0.368
	Female	4.30	0.94							4.35	1.03						
Fear of making mistakes during work	Male	2.67	1.32	1.781	*p*=0.183	6.063	81.651	*p<0.0001**	-1.616, -0.818	2.26	1.29	3.185	*p*=0.076	4.838	87.363	*p<0.0001**	-1.453, -0.607
	Female	3.88	1.18							3.29	1.42						
Fear of malpractice litigation	Male	2.06	1.32	1.939	*p*=0.165	3.351	86.606	*p<0.001**	-1.140, -0.291	1.78	1.11	7.838	*p*=0.006	3.682	97.308	*p<0.0001**	-1.074, 0.322
	Female	2.77	1.38							2.48	1.34						
Financial difficulties	Male	2.71	1.30	1.732	*p*=0.189	2.890	76.114	*p<0.0031**	-0.992, -0.183	2.46	1.30	0.435	*p*=0.510	3.710	82.347	*p<0.0001**	-1.198, -0.362
	Female	3.29	1.19							3.24	1.36						
Fulfilment of requirements in practice	Male	2.72	1.32	3.767	*p*=0.053	3.359	75.202	*p<0.001**	-1.068, -0.273	2.39	1.28	0.279	*p*=0.598	3.647	84.673	*p<0.0001**	-1.163, -0.342
	Female	3.39	1.11							3.14	1.34						
High expectations from animal owners	Male	3.11	1.25	1.869	*p*=0.173	4.106	79.516	*p<0.0001**	-1.170, -0.406	2.44	1.26	1.503	*p*=0.222	4.317	89.703	*p<0.0001**	-1.279, -0.473
	Female	3.90	1.12							3.32	1.36						
Lack of participation in decision making	Male	1.89	1.32	0.453	*p*=0.501	3.045	75.601	*p<0.0031**	-1.107, -0.232	1.73	1.02	10.197	*p<0.0031**	3.045	86.696	*p*=0.009	-0.935, -0.196
	Female	2.56	1.36							2.29	1.33						
Negative comments, bad reviews	Male	3.08	1.36	1.912	*p*=0.168	3.580	75.427	*p<0.001**	-1.159, -0.330	2.63	1.21	0.111	*p*=0.739	5.780	84.610	*p<0.0001**	-1.483, -0.724
	Female	3.82	1.18							3.74	1.21						
Practice management business, not enough profit	Male	2.92	1.35	0.074	*p*=0.785	0.060	86.986	*p*=0.952	-0.451, 0.424	2.56	1.29	0.050	*p*=0.824	1.132	82.692	*p*=0.261	-0.675, 0.185
	Female	2.93	1.31							2.81	1.31						
Professional competition	Male	2.25	1.27	0.465	*p*=0.496	0.943	89.463	*p*=0.348	-0.590, 0.210	1.90	1.07	2.352	*p*=0.127	1.770	97.210	*p*=0.080	-0.664, 0.038
	Female	2.44	1.31							2.22	1.24						
There is little social support	Male	2.40	1.26	0.123	*p*=0.727	2.791	87.428	*p*=0.007	-0.961, -0.162	2.12	1.17	6.559	*p*=0.011	2.908	94.158	*p*=0.009	-0.967, -0.182
	Female	2.96	1.31							2.69	1.39						

An asterisk (*) denotes a statistically significant association (Bonferroni-adjusted $p \leq 0.0031$).
Responses were given on a 5-point Likert scale, where 1 = strongly disagree, 2 = disagree, 3 = neither agree nor disagree, 4 = agree, 5 = strongly agree.

Supplementary Table S4. Working-hour differences in the assessment of work-related stressors and strain-related factors among Hungarian veterinarians (*n* = 231).

Stressor-related items (*n*=16)	Working hours	Effect on veterinary work							Effect on personal life						
		M	SD	Pearson's chi-square (χ2) test			Cramer's *V*		M	SD	Pearson's chi-square (χ2) test			Cramer's *V*	
				χ^2	*df*	*p* value	Value	*p* value			χ^2	*df*	*p* value	Value	*p* value
Burnout	Weekly <40 hours	3.63	1.24	3.155	8	*p*=0.924	0.087	*p*=0.924	3.59	1.40	3.150	8	*p*=0.925	0.087	*p*=0.925
	Weekly >40 hours	3.64	1.26						3.59	1.37					
Complaints from dissatisfied animal owners	Weekly <40 hours	3.57	1.26	7.826	8	*p*=0.451	0.132	*p*=0.451	3.22	1.32	3.876	8	*p*=0.868	0.093	*p*=0.868
	Weekly >40 hours	3.60	1.23						3.16	1.39					
Daily contact with staff	Weekly <40 hours	2.23	1.32	16.757	8	*p*=0.033	0.192	*p*=0.033	2.29	1.42	8.885	8	*p*=0.352	0.141	*p*=0.352
	Weekly >40 hours	2.55	1.32						2.61	1.43					
Ethical challenges	Weekly <40 hours	3.14	1.18	8.734	8	*p*=0.365	0.140	*p*=0.365	2.55	1.24	10.988	8	*p*=0.202	0.158	*p*=0.202
	Weekly >40 hours	2.98	1.30						2.66	1.27					
Euthanasia	Weekly <40 hours	2.71	1.30	7.941	8	*p*=0.429	0.135	*p*=0.429	2.30	1.31	5.611	8	*p*=0.691	0.115	*p*=0.691
	Weekly >40 hours	2.56	1.25						2.50	1.29					
Fatigue, emotional exhaustion	Weekly <40 hours	4.11	1.06	3.493	8	*p*=0.900	0.087	*p*=0.900	4.11	1.19	3.021	8	*p*=0.933	0.081	*p*=0.933
	Weekly >40 hours	4.19	1.07						4.22	1.16					
Fear of making mistakes during work	Weekly <40 hours	3.65	1.30	7.334	8	*p*=0.501	0.127	*p*=0.501	3.04	1.47	7.175	8	*p*=0.518	0.128	*p*=0.518
	Weekly >40 hours	3.52	1.34						3.05	1.46					
Fear of malpractice litigation	Weekly <40 hours	2.60	1.41	9.864	8	*p*=0.275	0.151	*p*=0.275	2.31	1.34	15.287	8	*p*=0.054	0.191	*p*=0.054
	Weekly >40 hours	2.55	1.37						2.26	1.24					
Financial difficulties	Weekly <40 hours	3.05	1.22	10.466	8	*p*=0.234	0.153	*p*=0.234	2.99	1.40	9.067	8	*p*=0.337	0.142	*p*=0.337
	Weekly >40 hours	3.25	1.23						3.09	1.36					
Fulfilment of requirements in practice	Weekly <40 hours	3.19	1.22	2.972	8	*p*=0.936	0.080	*p*=0.936	2.88	1.42	7.920	8	*p*=0.441	0.133	*p*=0.441
	Weekly >40 hours	3.28	1.16						3.04	1.27					
High expectations from animal owners	Weekly <40 hours	3.70	1.22	7.140	8	*p*=0.522	0.126	*p*=0.522	3.02	1.39	5.610	8	*p*=0.691	0.111	*p*=0.691
	Weekly >40 hours	3.72	1.19						3.23	1.40					
Lack of participation in decision making	Weekly <40 hours	2.37	1.37	8.826	8	*p*=0.357	0.143	*p*=0.357	2.06	1.31	24.434	8	*p<0.0031**	0.242	*p<0.0031**
	Weekly >40 hours	2.45	1.37						2.25	1.22					
Negative comments, bad reviews	Weekly <40 hours	3.62	1.27	11.312	8	*p*=0.185	0.158	*p*=0.185	3.48	1.30	4.191	8	*p*=0.839	0.097	*p*=0.839
	Weekly >40 hours	3.69	1.24						3.46	1.31					
Practice management business, not enough profit	Weekly <40 hours	2.87	1.30	5.084	8	*p*=0.749	0.116	*p*=0.749	2.67	1.30	11.256	8	*p*=0.188	0.173	*p*=0.188
	Weekly >40 hours	2.99	1.36						2.76	1.31					
Professional competition	Weekly <40 hours	2.33	1.32	13.277	8	*p*=0.103	0.172	*p*=0.103	2.06	1.22	9.715	8	*p*=0.286	0.149	*p*=0.286
	Weekly >40 hours	2.41	1.25						2.18	1.16					
There is little social support	Weekly <40 hours	2.72	1.30	12.081	8	*p*=0.148	0.164	*p*=0.148	2.51	1.41	17.031	8	*p*=0.030	0.198	*p*=0.030
	Weekly >40 hours	2.93	1.32						2.60	1.30					

An asterisk (*) denotes a statistically significant association (Bonferroni-adjusted $p \leq 0.0031$).
Responses were given on a 5-point Likert scale, where 1 = strongly disagree, 2 = disagree, 3 = neither agree nor disagree, 4 = agree, 5 = strongly agree.

Supplementary Table S5. Job-position differences in the assessment of work-related stressors and strain-related factors among Hungarian veterinarians (n = 220).

**Stressor-related items (n=16)**	**Position**	**Effect on veterinary work**							**Effect on personal life**						
		**M**	**SD**	**Pearson's chi-square (χ2) test**			**Cramer's *V***		**M**	**SD**	**Pearson's chi-square (χ2) test**			**Cramer's *V***	
				**χ^2**	***df***	***p* value**	**Value**	***p* value**			**χ^2**	***df***	***p* value**	**Value**	***p* value**
Burnout	Owner/manager position	3.40	1.38	15.058	8	*p*=0.058	0.190	*p*=0.058	3.33	1.54	12.878	8	*p*=0.116	0.175	*p*=0.116
	Employee/non-managerial position	3.83	1.15						3.82	1.23					
Complaints from dissatisfied animal owners	Owner/manager position	3.36	1.28	7.767	8	*p*=0.457	0.131	*p*=0.457	2.85	1.30	16.684	8	*p*=0.034	0.192	*p*=0.034
	Employee/non-managerial position	3.78	1.17						3.45	1.29					
Daily contact with staff	Owner/manager position	2.11	1.28	13.232	8	*p*=0.104	0.171	*p*=0.104	2.04	1.26	17.188	8	*p*=0.028	0.197	*p*=0.028
	Employee/non-managerial position	2.58	1.34						2.71	1.47					
Ethical challenges	Owner/manager position	2.82	1.24	13.205	8	*p*=0.105	0.172	*p*=0.105	2.18	1.15	19.096	8	*p*=0.014	0.209	*p*=0.014
	Employee/non-managerial position	3.20	1.20						2.88	1.24					
Euthanasia	Owner/manager position	2.38	1.29	16.094	8	*p*=0.041	0.192	*p*=0.041	2.09	1.22	8.388	8	*p*=0.396	0.140	*p*=0.396
	Employee/non-managerial position	2.76	1.23						2.54	1.33					
Fatigue, emotional exhaustion	Owner/manager position	3.98	1.14	8.957	8	*p*=0.346	0.139	*p*=0.346	4.04	1.25	12.760	8	*p*=0.120	0.167	*p*=0.120
	Employee/non-managerial position	4.26	0.96						4.29	1.07					
Fear of making mistakes during work	Owner/manager position	3.12	1.32	34.522	8	*p<0.0001**	0.276	*p<0.0001**	2.71	1.44	19.227	8	*p*=0.014	0.209	*p*=0.014
	Employee/non-managerial position	3.95	1.15						3.32	1.39					
Fear of malpractice litigation	Owner/manager position	2.18	1.24	20.826	8	*p*=0.007	0.220	*p*=0.007	2.05	1.20	15.031	8	*p*=0.059	0.189	*p*=0.059
	Employee/non-managerial position	2.92	1.41						2.53	1.33					
Financial difficulties	Owner/manager position	2.95	1.23	7.019	8	*p*=0.535	0.125	*p*=0.535	2.78	1.37	7.331	8	*p*=0.501	0.127	*p*=0.501
	Employee/non-managerial position	3.27	1.23						3.17	1.36					
Fulfilment of requirements in practice	Owner/manager position	2.95	1.24	9.321	8	*p*=0.316	0.142	*p*=0.316	2.66	1.33	10.015	8	*p*=0.264	0.149	*p*=0.264
	Employee/non-managerial position	3.38	1.15						3.18	1.34					
High expectations from animal owners	Owner/manager position	3.37	1.24	14.026	8	*p*=0.081	0.176	*p*=0.081	2.84	1.36	7.684	8	*p*=0.465	0.130	*p*=0.465
	Employee/non-managerial position	3.95	1.11						3.31	1.36					
Lack of participation in decision making	Owner/manager position	1.79	1.17	31.789	8	*p<0.0001**	0.272	*p<0.0001**	1.72	1.07	23.581	8	*p<0.0031**	0.238	*p<0.0031**
	Employee/non-managerial position	2.79	1.37						2.45	1.36					
Negative comments, bad reviews	Owner/manager position	3.34	1.33	16.765	8	*p*=0.033	0.192	*p*=0.033	3.05	1.30	17.049	8	*p*=0.030	0.195	*p*=0.030
	Employee/non-managerial position	3.83	1.16						3.74	1.21					
Practice management business, not enough profit	Owner/manager position	3.25	1.31	14.448	8	*p*=0.071	0.196	*p*=0.071	3.08	1.24	21.207	8	*p*=0.007	0.237	*p*=0.007
	Employee/non-managerial position	2.61	1.25						2.46	1.26					
Professional competition	Owner/manager position	2.21	1.22	5.846	8	*p*=0.664	0.114	*p*=0.664	1.92	1.10	8.464	8	*p*=0.389	0.139	*p*=0.389
	Employee/non-managerial position	2.52	1.34						2.30	1.24					
There is little social support	Owner/manager position	2.39	1.21	19.112	8	*p*=0.014	0.207	*p*=0.014	2.24	1.23	9.670	8	*p*=0.289	0.149	*p*=0.289
	Employee/non-managerial position	3.07	1.29						2.77	1.39					

An asterisk (*) denotes a statistically significant association (Bonferroni-adjusted $p \leq 0.0031$).
Responses were given on a 5-point Likert scale, where 1 = strongly disagree, 2 = disagree, 3 = neither agree nor disagree, 4 = agree, 5 = strongly agree.

Supplementary Table S6. Holiday-duration differences in the assessment of work-related stressors and strain-related factors among Hungarian veterinarians (n = 236).

Stressor-related items (n=16)	Holidays	Effect on veterinary work							Effect on personal life						
		M	SD	Pearson's chi-square (χ2) test			Cramer's *V*		M	SD	Pearson's chi-square (χ2) test			Cramer's *V*	
				χ^2	*df*	*p* value	Value	*p* value			χ^2	*df*	*p* value	Value	*p* value
Burnout	<14 days per year	3.93	1.16						4.09	1.30					
	14-28 days per year	3.58	1.23	8.600	8	*p*=0.377	0.14	*p*=0.377	3.53	1.37	12.843	8	*p*=0.117	0.175	*p*=0.117
	>28 days per year	3.53	1.36						3.33	1.42					
Complaints from dissatisfied animal owners	<14 days per year	3.69	1.24						3.29	1.47					
	14-28 days per year	3.66	1.23	4.020	8	*p*=0.855	0.10	*p*=0.855	3.39	1.32	18.621	8	*p*=0.017	0.203	*p*=0.017
	>28 days per year	3.36	1.29						2.64	1.14					
Daily contact with staff	<14 days per year	2.45	1.50						2.57	1.61					
	14-28 days per year	2.29	1.27	8.592	8	*p*=0.378	0.14	*p*=0.378	2.38	1.36	7.276	8	*p*=0.507	0.128	*p*=0.507
	>28 days per year	2.45	1.32						2.42	1.43					
Ethical challenges	<14 days per year	3.15	1.24						2.65	1.27					
	14-28 days per year	3.10	1.28	3.606	8	*p*=0.891	0.09	*p*=0.891	2.69	1.33	8.341	8	*p*=0.401	0.138	*p*=0.401
	>28 days per year	3.00	1.15						2.42	1.05					
Euthanasia	<14 days per year	2.75	1.25						2.44	1.30					
	14-28 days per year	2.54	1.26	3.171	8	*p*=0.923	0.09	*p*=0.923	2.39	1.31	0.512	8	*p*=0.999	0.035	*p*=0.999
	>28 days per year	2.79	1.30						2.33	1.30					
Fatigue, emotional exhaustion	<14 days per year	4.37	0.83						4.35	0.93					
	14-28 days per year	4.14	1.10	11.203	8	*p*=0.190	0.16	*p*=0.190	4.24	1.18	11.698	8	*p*=0.165	0.159	*p*=0.165
	>28 days per year	3.98	1.10						3.83	1.28					
Fear of making mistakes during work	<14 days per year	3.90	1.31						3.53	1.46					
	14-28 days per year	3.59	1.29	13.739	8	*p*=0.089	0.17	*p*=0.089	3.06	1.47	14.170	8	*p*=0.077	0.179	*p*=0.077
	>28 days per year	3.33	1.37						2.60	1.32					
Fear of malpractice litigation	<14 days per year	2.85	1.49						2.56	1.37					
	14-28 days per year	2.60	1.37	8.843	8	*p*=0.356	0.14	*p*=0.356	2.30	1.39	17.464	8	*p*=0.026	0.204	*p*=0.026
	>28 days per year	2.35	1.35						2.09	1.01					
Financial difficulties	<14 days per year	3.02	1.38						2.85	1.40					
	14-28 days per year	3.18	1.24	5.393	8	*p*=0.715	0.11	*p*=0.715	3.12	1.40	7.176	8	*p*=0.518	0.126	*p*=0.518
	>28 days per year	3.24	1.11						3.12	1.34					
Fulfilment of requirements in practice	<14 days per year	3.44	1.18						3.27	1.36					
	14-28 days per year	3.28	1.19	6.068	8	*p*=0.640	0.11	*p*=0.640	3.00	1.36	12.094	8	*p*=0.147	0.164	*p*=0.147
	>28 days per year	2.94	1.16						2.59	1.27					
High expectations from animal owners	<14 days per year	3.98	1.10						3.45	1.46					
	14-28 days per year	3.77	1.19	12.286	8	*p*=0.139	0.17	*p*=0.139	3.22	1.39	19.634	8	*p*=0.012	0.208	*p*=0.012
	>28 days per year	3.35	1.25						2.60	1.18					
Lack of participation in decision making	<14 days per year	2.61	1.45						2.50	1.47					
	14-28 days per year	2.36	1.34	5.792	8	*p*=0.671	0.12	*p*=0.671	2.11	1.28	5.919	8	*p*=0.656	0.119	*p*=0.656
	>28 days per year	2.42	1.42						2.09	1.18					
	<14 days per year	3.90	1.32	8.187	8	*p*=0.415	0.13	*p*=0.415	3.44	1.54	21.384	8	*p*=0.006	0.218	*p*=0.006

Negative comments, bad reviews	14-28 days per year	3.66	1.23						3.63	1.21					
	>28 days per year	3.42	1.25						3.17	1.23					
Practice management business, not enough profit	<14 days per year	3.09	1.46	11.115	8	*p*=0.195	0.17	*p*=0.195	2.93	1.50	10.820	8	*p*=0.212	0.169	*p*=0.212
	14-28 days per year	2.87	1.33						2.63	1.29					
	>28 days per year	2.90	1.14						2.78	1.10					
Professional competition	<14 days per year	2.63	1.41	10.199	8	*p*=0.251	0.15	*p*=0.251	2.33	1.34	8.192	8	*p*=0.415	0.137	*p*=0.415
	14-28 days per year	2.25	1.26						2.11	1.16					
	>28 days per year	2.50	1.30						2.00	1.17					
There is little social support	<14 days per year	2.91	1.35	8.164	8	*p*=0.418	0.14	*p*=0.418	2.98	1.50	16.543	8	*p*=0.035	0.195	*p*=0.035
	14-28 days per year	2.90	1.31						2.49	1.38					
	>28 days per year	2.59	1.30						2.38	1.10					

An asterisk (*) denotes a statistically significant association (Bonferroni-adjusted $p \leq 0.0031$).

Responses were given on a 5-point Likert scale, where 1 = strongly disagree, 2 = disagree, 3 = neither agree nor disagree, 4 = agree, 5 = strongly agree.
